# Multicenter performance evaluation and reference range determination of a new one‐stage factor VIII assay

**DOI:** 10.1002/jcla.24294

**Published:** 2022-03-11

**Authors:** Anna E. Lowe, Robert Jones, Steve Kitchen, Ulrich Geisen, Gergely Rozsnyai, Petra Jilma, Peter Quehenberger

**Affiliations:** ^1^ Sheffield Haemophilia and Thrombosis Centre Sheffield UK; ^2^ Institute for Clinical Chemistry and Laboratory Medicine Medical Center – University of Freiburg Faculty of Medicine University of Freiburg Freiburg Germany; ^3^ Roche Diagnostics International Ltd Rotkreuz Switzerland; ^4^ 27271 Department of Laboratory Medicine Medical University of Vienna Vienna Austria

**Keywords:** biological assay, diagnostic tests, factor VIII, hemophilia A, reference ranges

## Abstract

**Introduction:**

We conducted a multicenter evaluation of a new one‐stage factor VIII (FVIII) assay (Roche Diagnostics), intended for the quantitative assessment of FVIII activity. We evaluated the analytical performance of the FVIII assay on the cobas t 711 analyzer.

**Methods:**

Experiments performed at three laboratories used 3.2% citrated residual or commercially purchased plasma samples. Five human plasma pools and two controls were used to determine assay within‐run and within‐laboratory precision, and total reproducibility; coefficients of variation (CVs) and/or standard deviations (SDs) were calculated. Lot‐to‐lot variability and method comparison (vs Coagulation FVIII Deficient Plasma/Dade Actin FS Activated PTT reagent/Standard Human Plasma Calibrator on the Sysmex CS‐5100 analyzer; Siemens Healthineers) were evaluated by Passing–Bablok and Deming regression, respectively, and Pearson's *r* calculated. Assay‐specific reference range was determined using 199 fresh plasma samples from healthy adults, not receiving anticoagulants.

**Results:**

Across sites, SDs for repeatability were 0.016–0.046 for samples with ≤1.0 international units (IU)/dL FVIII activity; CVs were 0.9%–3.8% for samples with >1.0 IU/dl activity. Among samples with mean FVIII activity 0.344–133 IU/dl, good intermediate precision (SD 0.020 for samples with 0.344 IU/dl activity; CV 1.8%–4.7%) and good total reproducibility (CV 2.0%–13.3%) were observed. The FVIII assay showed excellent lot‐to‐lot variability (Pearson's *r* = .999) and good correlation with the comparator assay (Pearson's *r* = .993–.996). The reference range for FVIII activity was 82.2−218.0 IU/dl.

**Conclusion:**

The one‐stage FVIII assay demonstrated robust analytical performance on the cobas t 711 analyzer, supporting its use in routine laboratory practice.

## Graphical abstract

We conducted a multicenter evaluation of a new one‐stage factor VIII (FVIII) assay (Roche Diagnostics), intended for the quantitative assessment of FVIII activity. The new one‐stage FVIII assay demonstrated robust analytical performance on the cobas t 711 analyzer, supporting its use in routine laboratory practice.

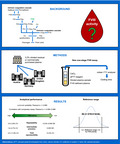

## 1 INTRODUCTION

Factor VIII (FVIII) is a multi‐domain glycoprotein that is synthesized in the liver.^1^, ^2^ As an essential blood coagulation protein required for normal hemostasis, FVIII circulates in plasma at normal concentrations of around 50–150 international units (IU)/dl in a non‐covalent complex with von Willebrand factor.^3^, ^4^ One of several essential factors of the intrinsic coagulation cascade, FVIII is activated to factor VIIIa via limited proteolysis by thrombin and then acts as a cofactor to activated factor IX, during the activation of factor X to factor Xa, ultimately leading to clot formation.^4^ Deficiency of or dysfunctional FVIII leads to a bleeding diathesis of varying severity, depending on FVIII levels and/or activity. Congenital FVIII deficiency, also known as hemophilia A, accounts for 80%–85% of hemophilia cases.^5^ Clinical manifestations of hemophilia A include spontaneous bleeding, particularly into joints and soft tissue, and excessive bleeding as a result of trauma or surgery.^6^, ^7^ FVIII deficiency may also be acquired,^8^ for example, due to the development of anti‐FVIII auto‐antibodies.^9^ In addition, FVIII deficiency is observed in some types of von Willebrand disease.^10^, ^11^ Conversely, elevated levels of FVIII increase the risk of first and recurrent venous thromboembolism,^12^, ^13^ underscoring the complex role of this glycoprotein in hemostasis.

FVIII in the form of recombinant or virus‐inactivated plasma‐derived clotting factor concentrate is a well‐established treatment for hemophilia A.^5^ Prophylactic FVIII replacement therapy, that is, regular rather than episodic administration, is the standard of care in patients with severe hemophilia A and some patients with moderate disease,^5^ providing safe and effective prevention of bleeding and preservation of joint health.^14^

According to recent World Federation of Hemophilia guidelines,^5^ a diagnosis of hemophilia A rests on three key features: (i) understanding the clinical features of the condition and appropriateness of a clinical diagnosis; (ii) use of screening tests, such as activated partial thromboplastin time (aPTT) and prothrombin time (PT) or platelet function tests, to identify potential causes of bleeding—a prolonged aPTT in conjunction with a normal PT and platelet count suggests the possible presence of hemophilia; and (iii) confirmation of appropriate diagnosis by a functional coagulation assay demonstrating FVIII deficiency (and exclusion of a differential diagnosis, such as von Willebrand disease).^15^ Coagulation assays are also used to determine the severity of hemophilia and monitor coagulation status before surgery, and for dose optimization during prophylactic treatment.

The aPTT assay can be used in combination with FVIII‐deficient plasma to quantify FVIII activity. One‐stage aPTT‐based assays are the most widely used functional coagulation assays in clinical laboratories.^15^ The assay evaluates the ability of patient plasma to correct the aPTT of a FVIII‐depleted plasma sample to a normal value, compared with a standard calibrator plasma.^16^ Key variables affecting assay performance include the instrument on which the assay is performed and the type of contact activator present in the aPTT reagent. Sensitivity to coagulation factor deficiencies varies across different aPTT reagents, while the majority can reliably detect moderate and severe hemophilia, not all are sufficient to detect mild hemophilia.^15^

Here, we present the results of a multicenter evaluation of a new one‐stage FVIII aPTT‐based assay (Roche Diagnostics International Ltd)^17^ intended for the quantitative assessment of FVIII activity. Our aim was to evaluate the analytical performance of the new assay on the cobas t 711 analyzer (Roche Diagnostics), a fully automated, high‐throughput, discrete coagulation instrument for the in vitro qualitative and quantitative determination of coagulation analytes in human plasma.^18^, ^19^

## 2 MATERIALS AND METHODS

### 2.1 Study design

This was a multicenter, non‐interventional, observational study conducted between November 2018 and June 2019, which was performed at three sites: University Hospital of Freiburg (Freiburg, Germany), Vienna General Hospital (Vienna, Austria), and Royal Hallamshire Hospital (Sheffield, United Kingdom [UK]).

The study was divided into two phases (Figure S1). An initial familiarization phase consisted of a within‐run precision test followed by an interlaboratory ring trial. Following the achievement of within‐run acceptance criteria, the ring trial ensured all sites produced similar results for measured quality control (QC) samples. The main study was performed in the second phase, comprising precision analysis and method comparison of the new one‐stage FVIII assay on the fully automated cobas t 711 analyzer (Roche Diagnostics) versus a commercially available comparator assay (Coagulation Factor VIII Deficient Plasma, Dade Actin FS Activated PTT Reagent, and Standard Human Plasma Calibrator, performed on the Sysmex CS‐5100 analyzer; Siemens Healthineers).^20^ Assay reproducibility, lot‐to‐lot variability, and the assay reference range were assessed in the second phase.

Independent ethics committee approval for the use of samples in Vienna was acquired from the Ethics Committee of the Medical University of Vienna, Austria (approval no. EK 1741/2017); the study protocol was submitted to the Austrian Agency for Health and Food Security. Approval was also granted from the Ethics Committee of the University of Freiburg (approval no. 10010/17). Study‐specific approval was not required for the use of anonymized residual samples in the UK. The study was performed according to the latest version of the Declaration of Helsinki and International Conference on Harmonisation Good Clinical Practice guidelines.

### 2.2 Determination of FVIII activity

FVIII activity was assessed using the new one‐stage FVIII assay on the cobas t 711 analyzer,^17^ methodology briefly as follows: FVIII activity was made rate‐limiting by diluting plasma samples (using imidazole buffer, pH 7.4; Roche Diagnostics) and adding the diluted samples to immunodepleted FVIII‐deficient human plasma (containing <1% of normal FVIII level); all other coagulation factors were present at levels typically >50% of normal. The aPTT reagent (aPTT; Roche Diagnostics; 600T, material number: 07153589190) used for the assay is lupus insensitive and contains ellagic acid as a surface activator and purified soy phosphatides. Resultant clotting times were interpreted using a calibration curve, obtained with dilutions of a calibrator plasma (Global Cal; Roche Diagnostics) mixed with FVIII‐deficient plasma; this calibrator method has been standardized against World Health Organization/National Institute for Biological Standards and Control standards.^21^ In aPTT‐based assays, the extent of correction of the aPTT is proportional to the activity of FVIII in the sample and is determined quantitatively from the calibration curve. Clots were detected using a photo‐optical method.

Each study site received two FVIII assay reagent lots: one main lot and one for lot‐to‐lot comparison. The Freiburg site received a third lot for reference range determination.

### 2.3 Samples and sample handling

Anonymized human 3.2% citrated plasma samples, both fresh and frozen to reflect routine laboratory practice, were used throughout the study. Samples were purchased from a commercial vendor for the reproducibility tests, and it was mandatory for all sites to use the same samples. The within‐run and within‐laboratory precision, total reproducibility, and method comparison tests used residual samples from the commercial vendor and/or residual samples from routine practice, comprising five human plasma pools (HPPs) covering the primary measuring range of the assay (0.200–150 IU/dl) and two controls (normal control and pathological control; three reagent lots; Roche Diagnostics). For the method comparison experiment, fresh and frozen samples were used at all sites.

The assay‐specific reference range was determined at the Freiburg site using 199 residual fresh samples (in three different reagent lots) from apparently healthy adult donors (≥18 years of age) who were not using anticoagulants. To confirm the reference range obtained from the fresh samples, these samples were then frozen, shipped, thawed, and tested at all three study sites. Prior to freezing, the samples were double‐spun, followed by flash freezing to below −60°C, and then shipped on dry ice and stored below −60°C for a maximum of 4–8 weeks; at the end of the study, all specimens were destroyed on site. An additional set of 121 anonymized frozen samples from apparently healthy adult donors (≥18 years of age) who were not using anticoagulants was analyzed at an internal Roche Diagnostics site (Penzberg, Germany) to confirm the reference range determined at the external study sites.

### 2.4 External quality control

An optional external quality assessment was performed to establish that the laboratories at each site yielded equivalent results and to assess their recovery of the provided samples in relation to other participants in the External Quality Assurance program. For this purpose, the UK National External Quality Assessment Scheme for Blood Coagulation (NEQAS, www.ukneqsbc.org) was selected. The Sheffield and Vienna sites participated in this program.

### 2.5 Data management and analyses

Assay results were directly captured by WinCAEv, a Code of Federal Regulations (Title 21, Part 11) compliant electronic data capture software developed and validated for Roche‐sponsored studies. Results entered offline were verified by the four‐eyes principle: every entry is checked against the source data by two individuals. Calculations of appropriate sample size were performed according to internal standard operating procedures, based on Clinical and Laboratory Standards Institute guidelines.^22^ Data were analyzed using WinCAEv (version 2.8.0) or BioWarp (version 1.2.4.7‐SR08) software.

Within‐run precision was assessed at each site using five HPPs covering the measuring range of the assay and two controls (*n* = 21 replicates per sample; one run per site) by calculating coefficients of variation (CVs) and standard deviations (SDs). Each site performed the measurements with their individual reagent and control lot using samples with ≤1.0 IU/dl or >1.0 IU/dl FVIII activity, supplied by Roche Diagnostics GmbH.

Intermediate (within‐laboratory) precision and total (across‐site) reproducibility were assessed at each site using five aliquots per sample per day, for five days, of the same five HPPs and two controls; CVs and SDs were calculated for each site and across the three sites.

Lot‐to‐lot variability and method comparison were conducted using >120 samples, determining the slope and intercept according to Passing–Bablok (lot‐to‐lot) or Deming (vs comparator reference) regression, and the correlation coefficient (Pearson's *r*).

The assay reference range of the new one‐stage FVIII assay on the cobas t 711 analyzer was determined using three reagent lots and 199 samples (assayed fresh, and after freezing) and taking the 2.5th and 97.5th percentiles as the upper and lower bounds of the range (i.e., the 95% confidence interval [CI], adopting a two‐sided, conservative, distribution‐free [rank‐based] approach). The assay reference range was also determined on the Sysmex CS‐5100 analyzer using frozen samples. Parametric descriptive statistics were used to calculate values for min, max, mean, and median. We employed an interquartile‐based method for the detection of any extreme values. All results were compared to predefined, assay‐specific acceptance criteria (Table S1).

## 3 RESULTS

All three sites passed the study familiarization experiment, which permitted each site to initiate the main phase of the study. To monitor the consistent quality of the results, daily QCs were performed using QC samples, tested at least twice per study day at all sites before and after measurement of experimental samples. The acceptance criterion was that control recovery needed to be within the defined target range of mean ±10%. Overall mean recovery ranged from 97.9% to 102% (Freiburg), 101% to 105% (Vienna), and 99.9% to 101% (Sheffield); CVs ranged from 2.2% to 3.2% (Freiburg), 2.0% to 2.8% (Vienna), and 2.4% to 3.3% (Sheffield). Thus, all QC measurements were within the predefined ranges.

### 3.1 Within‐run precision (repeatability)

The predefined acceptance criteria for within‐run precision were met (samples ≤1.0 IU/dl, SD ≤0.050; samples >1.0 IU/dl, CV ≤5.0%). The SD values ranged from 0.016 to 0.046 for samples with ≤1.0 IU/dl FVIII activity, and CV values ranged from 0.9% to 3.8% for samples with >1.0 IU/dl FVIII activity (Table 1).

**TABLE 1:** Within‐run precision (repeatability)^a^ of the new one‐stage FVIII assay on the cobas t 711 analyzer at each of the three study sites

Sample	Freiburg (Lot A)		Vienna (Lot B)		Sheffield (Lot C)	
	Mean FVIII activity, IU/dl (min, max)^b^	Within‐run precision (repeatability)^c^	Mean FVIII activity, IU/dl (min, max)^b^	Within‐run precision (repeatability)^c^	Mean FVIII activity, IU/dl (min, max)^b^	Within‐run precision (repeatability)^c^
HPP 1	0.375 (0.348, 0.472)	0.032 SD	0.343 (0.319, 0.380)	0.016 SD	0.302 (0.280, 0.341)	0.018 SD
HPP 2	1.09 (1.04, 1.15)	3.1	1.02 (0.930, 1.090)	3.8	0.896 (0.821, 0.978)	0.046 SD
HPP 3	5.05 (4.80, 5.26)	2.3	4.96 (4.63, 5.17)	3.0	4.99 (4.74, 5.21)	2.3
HPP 4	63.6 (61.9, 64.8)	1.1	65.4 (62.9, 68.2)	2.0	63.1 (61.1, 64.8)	1.6
HPP 5	129 (127, 132)	0.9	135 (130, 139)	1.9	127 (122, 134)	2.8
Control N	110 (106, 115)	1.7	109 (106, 114)	2.2	105 (101, 108)	2.0
Control P	25.8 (25.3, 26.5)	1.3	25.9 (25.0, 26.8)	2.3	25.0 (24.3, 25.7)	1.7

Abbreviations: Control N, normal control; Control P, pathological control; CV, coefficient of variation; FVIII, factor VIII; HPP, human plasma pool; IU, international units; max, maximum; min, minimum; SD, standard deviation.

^a^ Within‐run precision (repeatability) was assessed via one run per site (21 replicates per sample).

^b^ Samples with mean FVIII activity ≤1.0 IU/dL or >1.0 IU/dL were assessed by comparing the SD or CV, respectively, against acceptance criteria.

^c^ Values are CV% unless otherwise stated.

### 3.2 Intermediate precision and total reproducibility

Intermediate (within‐laboratory) precision and total (across‐site) reproducibility satisfied predefined acceptance criteria. Across all sites combined and for samples with mean FVIII activity of 0.344 to 133 IU/dl, good intermediate precision (SD 0.020 for samples with low [0.344 IU/dl] activity; CV 1.8% to 4.7% for samples with higher [>1.0 IU/dl] activity) and good total reproducibility (CV 2.0% to 13.3%) were observed and were within the predefined ranges (Table 2).

**TABLE 2:** Intermediate (within‐laboratory) precision and total (across‐site) reproducibility of the new one‐stage FVIII assay on the cobas t 711 analyzer across the three study sites

Sample	Site	Mean FVIII activity (IU/dl)^a^	Intermediate (within‐laboratory) precision^b^	Total (across‐site) reproducibility (CV%)
HPP 1	All	0.344	0.020 SD	13.3
	Freiburg	0.389	0.019 SD	
	Vienna	0.336	0.021 SD	
	Sheffield	0.307	0.021 SD	
HPP 2	All	1.04	4.7	11.9
	Freiburg	1.14	4.7	
	Vienna	1.05	4.8	
	Sheffield	0.917	0.044 SD	
HPP 3	All	5.08	3.1	3.9
	Freiburg	5.22	2.6	
	Vienna	4.98	3.9	
	Sheffield	5.02	2.8	
HPP 4	All	65.5	1.8	2.0
	Freiburg	66.1	1.4	
	Vienna	65.6	1.6	
	Sheffield	64.8	2.4	
HPP 5	All	133	1.9	2.1
	Freiburg	134	1.5	
	Vienna	135	2.2	
	Sheffield	132	1.9	
Control N	All	107	1.8	2.5
	Freiburg	109	1.7	
	Vienna	105	2.0	
	Sheffield	107	1.9	
Control P	All	25.5	2.2	2.2
	Freiburg	25.6	1.6	
	Vienna	25.5	2.1	
	Sheffield	25.5	2.7	

Abbreviations: Control N, normal control; Control P, pathological control; CV, coefficient of variation; FVIII, factor VIII; HPP, human plasma pool; IU, international units; SD, standard deviation.

^a^ Samples were assessed by comparing the SD or CV, as appropriate, against the following predefined acceptance criteria: intermediate precision, SD ≤0.060 for samples ≤1.0 IU/dl and CV ≤6.0% for samples >1.0 IU/dl (based on mean FVIII activity); total reproducibility, CV ≤25.0% (based on relative FVIII activity).

^b^ Values are CV% unless otherwise stated.

### 3.3 Lot‐to‐lot variability

**FIGURE 1:**
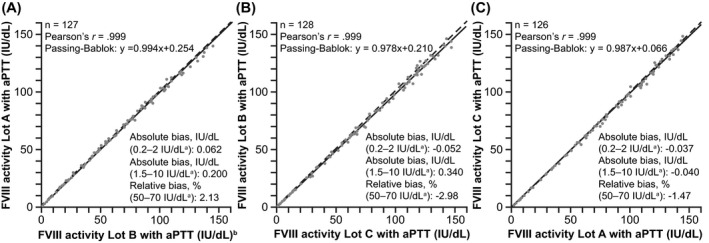
Lot‐to‐lot variability of the new one‐stage FVIII assay on the cobas t 711 analyzer at each of the three study sites: (A) Freiburg, (B) Vienna, and (C) Sheffield. ^a^Activity range. ^b^19 additional pairs outside the limits have not been used for calculation. aPTT, activated partial thromboplastin time; FVIII, factor VIII; IU, international units

Assessment of reagent lot‐to‐lot variability was performed at all sites; each site compared two different lots out of the three used in total. The new one‐stage FVIII assay showed low lot‐to‐lot variability (Pearson's *r* =.999 for all three sites; Figure 1).

### 3.4 Method comparison

**FIGURE 2:**
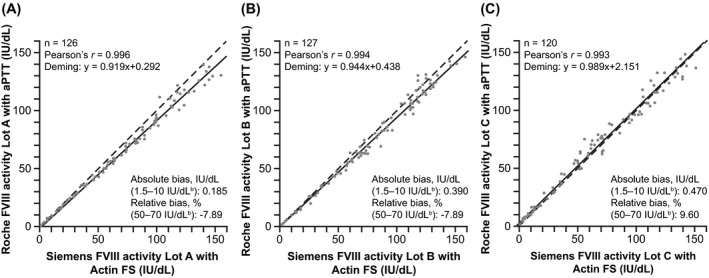
Method comparison of the new one‐stage FVIII assay on the cobas t 711 analyzer vs reference comparator assay^a^ at each of the three study sites: (A) Freiburg, (B) Vienna, and (C) Sheffield. ^a^Siemens Sysmex CS‐5100 analyzer and Coagulation Factor VIII Deficient Plasma in combination with Dade Actin FS Activated PTT reagent. ^b^Activity range. aPTT, activated partial thromboplastin time; FVIII, factor VIII; IU, international units

Method comparisons were conducted at each site across the primary measuring range of both the new one‐stage FVIII assay on the cobas t 711 analyzer and reference comparator assay on the Sysmex CS‐5100 analyzer. Good agreement (Pearson's *r*) between the new one‐stage FVIII assay and comparator assay was observed at each site: Freiburg, *r* =.996; Vienna, *r* =.994; Sheffield, *r* =.993 (Figure 2).

### 3.5 Assay reference range

**FIGURE 3:**
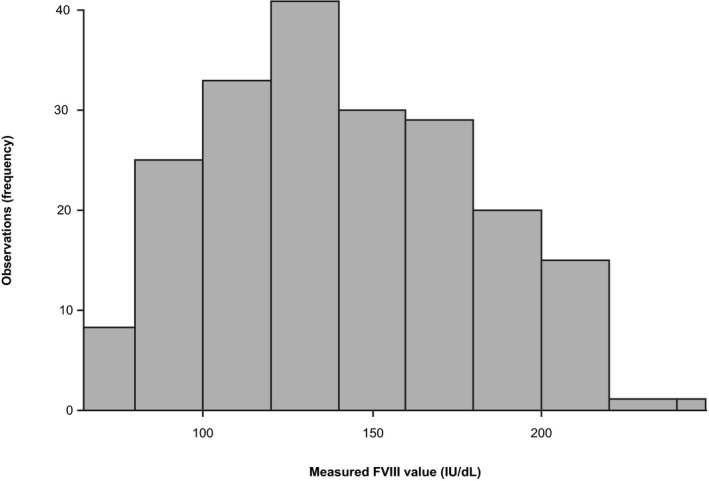
Distribution of factor VIII reference values measured at the Freiburg site in 199 fresh samples (three reagent lots) from apparently healthy adult donors (≥18 years of age) who were not using anticoagulants. FVIII, factor VIII; IU, international units

Among apparently healthy individuals, FVIII activity ranged from 82.2 to 218.0 IU/dl (2.5–97.5th percentiles of the assay results) in fresh samples and from 79.8 to 211.0 IU/dl (2.5–97.5th percentiles of the assay results) in frozen samples. The distribution of measured FVIII values in fresh samples at the Freiburg site is shown in Figure 3; no outlier/extreme values were observed or excluded. As the determined reference range of 82.2 to 218.0 IU/dl in fresh samples was higher than the expected normal range (50 to 150 IU/dl),^3^ an additional set of 121 anonymized frozen samples from apparently healthy donors was measured at an internal Roche site (Penzberg, Germany) and the determined reference range (75 to 210 IU/dl) confirmed the initial measurements.

Testing of frozen samples using the Sysmex CS‐5100 analyzer generated a similar reference range (81 to 215 IU/dl) to that obtained with the cobas t 711 analyzer.

### 3.6 External quality control

The NEQAS results were near‐identical for both participating sites (Sheffield and Vienna); both achieved performance grading “a,” indicating that the results were acceptable. The result was 6.54 IU/dl for Sheffield and 6.75 IU/dl for Vienna. The percentage deviation from the median result (7 IU/dl) for Sheffield and Vienna was −6.6% and −3.6%, respectively.

### 3.7 Adverse device events and protocol deviations

No adverse device events or protocol deviations were observed during the study.

## 4 DISCUSSION

FVIII is an essential blood coagulation factor, the deficiency of which manifests as a bleeding phenotype of varying severity. Hence, robust and reliable laboratory measurement of FVIII is required to monitor levels, track response to treatment with FVIII substitution therapy, and guide appropriate dosing. Results from this multicenter, observational study conducted in Austria, Germany, and the UK demonstrate that the new one‐stage FVIII assay on the cobas t 711 analyzer has excellent repeatability (within‐run precision), intermediate (within‐laboratory) precision, total (across‐site) reproducibility, and lot‐to‐lot variability. Good correlation (Pearson's *r* =.993–.996) was observed with a commercially available comparator assay, and predefined acceptance criteria were satisfied for all analytical tests.

The reference range of the new one‐stage FVIII assay in fresh samples from apparently healthy individuals was determined as 82.2 to 218.0 IU/dl (2.5–97.5th percentiles of the assay results). While the present reference range was wider and higher compared with the frequently quoted normal range (50 to 150 IU/dl),^3^ repeat testing with additional samples from different sample sources resulted in an equivalent range. It should also be noted that the latest reported reference range for the comparator assay used herein (Siemens FVIII assay on the Siemens Atellica COAG 360 System) is 79.5 to 216.3 IU/dl,^23^ which is comparable with our data. Furthermore, testing of frozen samples in the present study using the Sysmex CS‐5100 analyzer generated a similar reference range (81 to 215 IU/dl) to that obtained with the cobas t 711 analyzer. Therefore, the wider range found in the current study may reflect a contemporary upward shift in the current “normal” range of 50 to 150 IU/dl.

FVIII activity levels can be measured with a one‐stage aPTT‐based or a two‐stage chromogenic factor activity assay. The one‐stage assay is the most common method used by clinical diagnostic laboratories to assess FVIII activity, in Europe and globally.^24^ This preference is attributable to various factors, including a heritage of long‐standing use, cost advantages, and a perception of chromogenic assays as less rapid and more technically complex. The United States Food and Drug Administration's preference that the potency labeling of FVIII replacement therapies should be established using one‐stage assays may have contributed to the current common laboratory practice of using one‐stage assays to measure clinical patient samples.^25^

Our findings demonstrated good total reproducibility of the new one‐stage FVIII assay across the three study sites: two of the five HPPs had measurements with CVs of <2.5% and all values were well within the predefined acceptance criteria (CV ≤25.0%). The ability of this assay to detect FVIII activities of ˂1.00 IU/dl, as was measured in HPP 1 at all sites and in HPP 2 at Sheffield (Table 2), has beneficial clinical implications for severe hemophilia and indicates a high level of accuracy and precision. The automated reconstitution of the assay reagent by the instrument also provides a technical advantage.

The strengths of this study include its multicenter and multinational design and the use of multiple parameters to assess analytical performance. The inclusion of the familiarization phase and the interlaboratory ring trial provides additional rigor. A key limitation was the use of anonymized samples, which prohibited assessment of the potential impact of any underlying comorbidities or concomitant medications on the accuracy of results.

In common with all one‐stage assays, the new one‐stage FVIII assay may not be appropriate for the measurement of FVIII activity induced by some extended half‐life recombinant clotting factors, which are emerging as prophylactic treatment options with advantages over standard products.^15^ With a heterogeneous structure and mode of action, extended half‐life factors show wide variation in activity levels between different one‐stage assays, and further evaluation studies are necessary to assess the existence or extent of this effect with the new one‐stage FVIII assay tested in the present paper.

In conclusion, the new one‐stage FVIII assay demonstrated robust analytical performance on the cobas t 711 analyzer, supporting its use in routine laboratory practice.

## CONFLICT OF INTEREST

AEL has acted as a paid speaker to Swedish Orphan Biovitrum and received funding on this occasion and has received travel reimbursement from Roche Diagnostics International Ltd for attending a symposium as a scientific poster presenter. SK has received consultancy and speaker fees from Roche Diagnostics International Ltd and Werfen. UG has received funding for a technician to carry out this work from Roche Diagnostics International Ltd, support for attending meetings, and/or travel from Takeda Pharma Vertrieb GmbH & Co. KG, Bayer Vital GmbH, and CSL Behring and received honoraria for participation in an Advisory Board for Roche Pharma AG. GR is an employee of Roche Diagnostics International Ltd. RJ, PJ, and PQ report no interests which might be perceived as posing a conflict or bias.

## AUTHOR CONTRIBUTIONS

SK and GR designed the research study. AEL, RJ, UG, GR, and PJ performed the study. SK, UG, GR, PJ, and PQ analyzed the data. All authors contributed to the writing and critical review of the paper and approved the final version for submission.

## Supporting information

Supplementary Material

## Data Availability

Qualified researchers may request access to individual anonymized patient‐level data through the clinical study data request platform (https://vivli.org/). Further details on Roche's criteria for eligible studies are available here: https://vivli.org/members/ourmembers/. For further details on Roche's Global Policy on the Sharing of Clinical Information and how to request access to related clinical study documents, see here: https://www.roche.com/research_and_development/who_we_are_how_we_work/clinical_trials/our_commitment_to_data_sharing.htm.
