# Supplementary material for: Multicenter performance evaluation and reference range determination of a new one‐stage factor VIII assay
Source: J Clin Lab Anal. 2022 Mar 11;36(4):e24294. doi: 10.1002/jcla.24294 (PMC8993620; doi:10.1002/jcla.24294)
Supplement: Supplementary file 1 — Supplementary Material [file JCLA-36-e24294-s001.docx]

**SUPPLEMENTARY MATERIALS**

**SUPPLEMENTARY TABLE 1** Pre-defined acceptance criteria used in this study to evaluate the analytical performance of the new one-stage FVIII assay on the cobas t 711 analyzer

| **Experiment/Test** | **Acceptance criteria** |
| --- | --- |
| Within-run precision (repeatability)^a^ | Samples ≤1.0 IU/dL: SD ≤0.050; Samples >1.0 IU/dL: CV ≤5.0% |
| Intermediate (within-laboratory) precision^a^ | Samples ≤1.0 IU/dL: SD ≤0.060; Samples >1.0 IU/dL: CV ≤6.0% |
| Reproducibility^b^ | Total (across-site): CV ≤25.0% |
| Lot-to-lot variability | Correlation (Pearson’s *r*): ≥.950  Slope (Passing-Bablok): 1.000±0.050 Bias: Median deviation between lots ≤±0.400 IU/dL (activity range 0.2–2 IU/dL) or ≤±1.00 IU/dL (activity range 1.5–10 IU/dL) absolute deviation, and ≤±10.0% relative deviation (activity range 50–70 IU/dL) |
| Method comparison vs reference | Correlation (Pearson’s *r*): ≥.900; Slope (Passing-Bablok): 1.000±0.100 Bias: Median deviation between cobas t 711 and reference instrument ≤1.00 IU/dL absolute deviation (activity range 1.5–10 IU/dL), and ≤15.0% relative deviation (activity range 50–70 IU/dL) |
| Reference range determination | 50–150 IU/dL^c^ |

^a^Based on mean FVIII activity.
^b^Based on relative FVIII activity.
^c^Indicated range based on previously reported data in the literature.^1^

Abbreviations: CV, coefficient of variation; FVIII, factor VIII; IU, international units; SD, standard deviation.

**SUPPLEMENTARY FIGURE 1** Study design schematic.


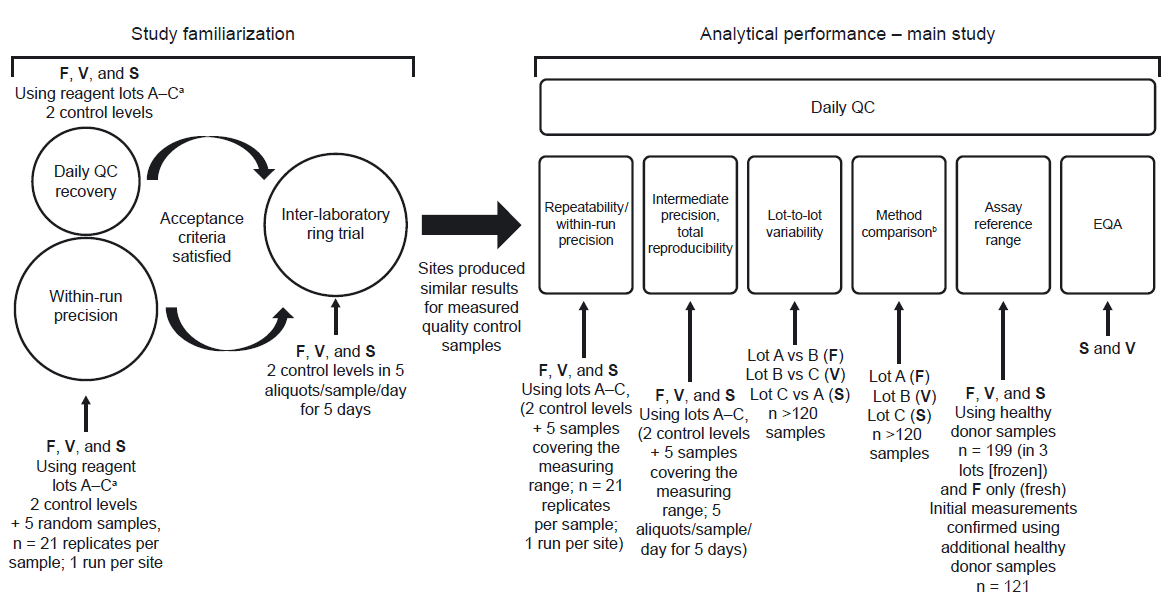

^a^Reagent lots A, B, and C each contained five human plasma samples (HPP 1−5) plus two controls (normal control and pathological control) as fresh/frozen 3.2% citrated samples.
^b^Using Siemens Sysmex CS-5100 analyzer and Siemens Coagulation Factor VIII Deficient Plasma (in combination with Actin FS) reagent as comparator.
EQA, External Quality Assurance; F, Freiburg; QC, quality control; S, Sheffield; V, Vienna.

# REFERENCES

1. Fijnvandraat K, Cnossen MH, Leebeek FW, Peters M. Diagnosis and management of haemophilia. *BMJ.* 2012;344:e2707.
